# Omics-Based Functional Fingerprinting of Nanoparticles in Cancer: Toward Predictive Nanomedicine

**DOI:** 10.3390/ijms27041960

**Published:** 2026-02-18

**Authors:** Serena Marchiò

**Affiliations:** 1 Department of Oncology, University of Turin, 10060 Candiolo, Italy; serena.marchio@unito.it; Tel.: +39-011-9933-3239; 2 Candiolo Cancer Institute, Fondazione del Piemonte per l’Oncologia—Istituto di Ricovero e Cura a Carattere Scientifico (FPO-IRCCS), 10060 Candiolo, Italy

**Keywords:** nanoparticle, precision medicine, cancer, transcriptomics, proteomics, metabolomics, single-cell omics, spatial omics, nanotoxicology, systems biology

## Abstract

Nanoparticles are widely explored in oncology as delivery platforms for cytotoxic drugs and molecularly defined therapeutic agents, including immunomodulators. While advances in nanomaterial engineering have enabled precise control over physicochemical properties, biological responses to nanoparticles remain difficult to predict and often diverge across experimental systems. Recent omics studies reveal that nanoparticle exposure induces coordinated cellular programs that extend beyond overt toxicity and are strongly shaped by delivery context, cellular state, and microenvironmental conditions. Importantly, these responses cannot be attributed solely to the payload, as nanocarriers themselves frequently engage stress, metabolic, and immune-related pathways, giving rise to non-additive and context-dependent effects. This Perspective proposes omics-based functional fingerprinting as a conceptual framework to interpret nanoparticle biology in cancer. Functional fingerprints are defined as integrated biological response states arising from nanocarrier–payload systems and resolving through transcriptomic, proteomic, metabolomic, and emerging single-cell or spatial approaches. By explicitly distinguishing carrier-dependent, payload-induced, and composite response programs, functional fingerprinting provides a means to reconcile heterogeneous observations and move beyond material-centered classification. Incorporating biological resolution and context awareness into nanoparticle profiling is expected to improve mechanistic interpretation, safety assessment, and the rational design of more predictive nanomedicine strategies.

## 1. Introduction

Nanoparticles are nanoscale modular systems composed of at least a carrier and an active ingredient (payload), which is concentrated, protected from degradation, and prevented from causing unwanted effects until it reaches its target site [1]. They are widely investigated in oncology as platforms for drug delivery, with the aim of improving pharmacokinetics, biodistribution, and therapeutic index [1,2]. Advances in nanomaterial synthesis have enabled increasingly precise control over size, shape, surface chemistry, and composition, reinforcing the expectation that rational carrier design can enhance drug performance and therapeutic selectivity [2,3]. Nevertheless, seminal and recent studies have highlighted a persistent gap between material optimization and biological outcome, emphasizing that delivery and efficacy cannot be inferred from physicochemical parameters alone [4,5].

In nanoparticle-based systems, the payload represents the intended source of biological activity. Its therapeutic effect typically relies on inducing cytotoxic or cytostatic stress in cancer cells, and its molecular impact depends on delivery efficiency, intracellular trafficking, release kinetics, and the functional state of the receiving cell [2]. Consequently, the same active agent can display distinct biological effects depending on how it is delivered [6,7], underscoring that payload behavior is context-dependent and shaped by the carrier and the cellular environment rather than being an intrinsic, fixed property of the drug.

By contrast, the nanocarrier is generally expected to be biologically neutral or biocompatible, serving primarily as a vehicle for payload delivery—a principle that underlies much of nanomedicine design [8,9]. However, a growing body of nanotoxicology and nano–bio interaction studies challenge this assumption. Omics-informed studies, particularly proteomic and metabolomic analyses, show that nanomaterials, even in the absence of active payloads, can elicit measurable cellular responses, including engagement of stress pathways, modulation of redox balance, interactions with proteins and membranes, and alterations in metabolic homeostasis [8,10,11].

Importantly, carrier-driven biological effects raise distinct concerns depending on cellular context. In non-malignant cells, nanoparticle exposure can result in off-target responses and toxicity, with implications for systemic safety. In cancer cells, exposure to nanocarriers alone can induce adaptive programs, such as proteostasis engagement, metabolic remodeling, and stress tolerance, that are difficult to predict from material descriptors and may influence therapeutic sensitivity and treatment outcomes [12,13,14]. These observations indicate that nanocarriers are not universally inert and that their biological activity can meaningfully shape cellular states relevant to therapy.

High-throughput omics technologies have been instrumental in exposing this complexity. Transcriptomic, proteomic, and metabolomic approaches reveal coordinated cellular programs activated by nanoparticle exposure, often detectable at subcytotoxic doses and independently of overt effects on viability or proliferation [13,14]. At the same time, these studies highlight substantial heterogeneity in experimental design: some focus on empty nanocarriers to assess intrinsic material effects, others analyze drug-loaded nanoparticles to evaluate efficacy, and only a subset explicitly compares empty carriers, free payloads, and loaded systems under aligned conditions. As a result, reported “nanoparticle-induced signatures” often conflate payload-driven pharmacological activity with carrier-associated biological perturbations [15,16].

**Figure 1:**
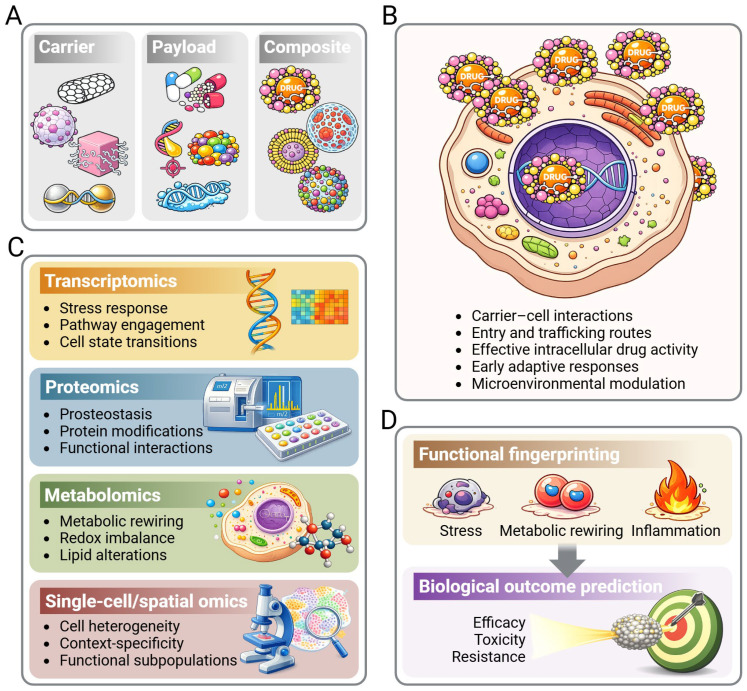
Omics-based functional fingerprinting of nanoparticle systems in cancer. (**A**) Nanoparticles are composed of distinct but interacting components, including the nanocarrier, the therapeutic payload, and their composite assembly. The effects of these elements are frequently non-additive and poorly predictable based on physicochemical properties or drug identity alone. (**B**) Upon exposure to cancer cells, nanoparticles engage a series of cell-centric processes, including carrier–cell interactions, cellular entry and intracellular trafficking routes, effective intracellular payload activity, early adaptive responses, and modulation by the microenvironment. (**C**) Multi-layered omics approaches provide complementary readouts of these state changes. Transcriptomics captures coordinated stress responses, pathway engagement, and cell state transitions; proteomics resolves proteostasis regulation, protein modifications, and functional interaction networks; metabolomics reveals metabolic rewiring, redox imbalance, and lipid alterations; and single-cell or spatial omics resolve the cellular heterogeneity, context specificity, and functional subpopulations that are obscured in bulk analyses. (**D**) Integration of these molecular layers defines a functional fingerprint, reflecting emergent cellular states such as stress adaptation, metabolic reprogramming, and inflammatory signaling. These fingerprints provide a framework to interpret and anticipate the biological outcomes of nanoparticle exposure, including therapeutic efficacy, toxicity, and resistance, thereby linking nanoparticle design to context-aware and more predictive nanomedicine strategies.

This Perspective argues that resolving this ambiguity is essential for progress in cancer nanomedicine. Failure to distinguish carrier- and payload-driven responses risks misinterpreting adaptive cellular states, obscuring both potential safety liabilities and context-dependent therapeutic opportunities. In parallel, the integration of high-throughput molecular profiling with nanotechnology has been increasingly explored across pharmaceutical and biomaterials sciences, highlighting the need to systematically connect nanoparticle physicochemical properties with multi-layered biological responses. These efforts reinforce the rationale for structured frameworks capable of organizing heterogeneous omics outputs into interpretable biological states, particularly in oncology where context-dependence is pronounced. The conceptual framework of omics-based functional fingerprinting proposed here is schematically summarized in Figure 1.

## 2. Omics as an Entry Point to Nanocarrier Functional Fingerprinting

Nanocarriers are often designed with the expectation of relative biological neutrality [9], yet accumulating evidence indicates that nanomaterials themselves can actively perturb cellular systems. Independent of payload, nanocarriers rapidly acquire a biological identity through adsorption of biomolecules, commonly referred to as the protein corona [17], and subsequently engage cell membranes, internalization pathways, and intracellular trafficking routes [18]. These processes, largely dictated by nanocarrier surface properties rather than by the payload, can trigger adaptive or stress-related cellular responses. Together, these observations position the nanocarrier as a biologically active component whose effects warrant independent characterization. This distinction between nanocarrier-driven, payload-driven, and composite response programs underlies the functional fingerprinting framework illustrated in Figure 1.

The convergence between nanotechnology and high-throughput molecular profiling has increasingly been described as “nano-omics,” reflecting efforts to systematically map nanoparticle–biological system interactions across multiple regulatory layers. While nano-omics emphasizes integrative characterization of nano–bio interfaces, the framework proposed here further resolves these interactions within cancer-specific contexts to clarify how distinct components shape emergent cellular states.

Omics-based studies have been particularly informative in revealing nanocarrier-driven responses that are not captured by conventional toxicity assays. Transcriptomic, proteomic, and metabolomic analyses consistently detect activation of coordinated cellular programs, encompassing oxidative stress, proteostasis, inflammatory signaling, and metabolic adaptation, following exposure to empty nanomaterials, including metallic, polymeric, lipid-based, and inorganic nanoparticle systems [12,19,20,21]. Beyond steady-state gene expression changes, nanoparticle exposure has also been associated with epigenetic remodeling and altered microRNA (miRNA) profiles, which may further modulate transcriptional programs and cellular state transitions. These regulatory layers contribute to the persistence, reversibility, or amplification of nanoparticle-induced responses, reinforcing the multidimensional nature of functional fingerprints. Importantly, such responses are often observed at doses that do not cause overt cytotoxicity, indicating that nanocarriers can induce functional state changes without compromising short-term viability [12,13]. Rather than being dismissed as background noise, these sub-clinical perturbations may act as biological priming events, subtly reshaping the baseline functional state of exposed cells. Such priming could influence subsequent responsiveness to therapeutic payloads, modulate stress thresholds, or alter adaptive capacity, thereby contributing to composite nanoparticle effects beyond immediate toxicity readouts. Conceptually, this shifts carrier-driven fingerprints from incidental perturbations to determinants of cellular trajectory.

Importantly, transcriptional alterations induced by nanocarriers or nanocarrier–payload systems may propagate to downstream proteomic and metabolic networks, influencing enzyme activity, signaling cascades, and adaptive stress pathways. Such multilayered modulation helps explain how apparently modest gene expression changes can translate into divergent functional outcomes across cellular contexts.

The biological impact of nanocarriers is strongly context-dependent. In non-malignant cells, carrier-induced stress responses may translate into off-target toxicity, with implications for systemic safety and therapeutic window. In cancer cells, by contrast, nanocarrier exposure can engage adaptive programs that intersect with established hallmarks of cancer, including altered metabolism, stress tolerance, and survival signaling [12,13,14]. Although these effects may be subtle, they can be biologically consequential, shaping how cancer cells respond to subsequent therapeutic challenges [12,22].

Nanocarrier-driven functional fingerprints are not uniform across materials or cell types. Variations in size, surface chemistry, surface charge, rigidity, structural configuration (including chiral arrangement), and protein corona composition influence uptake routes and intracellular fate, resulting in distinct molecular response patterns even among closely related nanomaterials [22,23]. This variability helps explain why physicochemical similarity does not necessarily translate into biological equivalence and underscores the limits of material-centered classification schemes [16,22].

From a functional fingerprinting perspective, nanocarrier-induced responses should be treated as a distinct analytical layer rather than as incidental perturbations. Omics profiling provides a systematic approach to capture these responses, enabling comparison across materials, cell types, and exposure conditions [13]. Such fingerprints can inform both safety and efficacy studies, by identifying carrier-associated liabilities and carrier-driven cellular states that may potentiate or attenuate payload activity, respectively [15,16].

Together, these observations indicate that nanocarriers are active modulators of cellular states. Explicitly defining nanocarrier-driven functional fingerprints is therefore a prerequisite for disentangling composite nanoparticle effects and for interpreting the biological consequences of drug-loaded nanotherapeutic systems.

## 3. Payload-Driven and Composite Functional Fingerprints

While nanocarrier-driven effects define an important baseline, the payload remains the primary source of intended therapeutic activity in most cancer nanomedicine applications. Cytotoxic and targeted agents are designed to perturb specific cellular processes, such as DNA replication, mitotic progression, signaling pathways, or metabolic dependencies, and their efficacy is commonly evaluated through phenotypic endpoints including cell death, growth inhibition, or tumor regression [2]. From a functional fingerprinting standpoint, these payload-driven effects correspond to molecular programs associated with target engagement and downstream stress responses.

However, payload activity cannot be fully understood independent of its delivery context. Encapsulation within nanocarriers can alter intracellular trafficking routes, subcellular localization, release kinetics, and exposure duration, thereby reshaping the molecular consequences of payload action. As a result, the functional fingerprint of a given drug delivered via nanoparticles may differ qualitatively from that induced by the same compound administered in free form, even when nominal doses and apparent efficacy are comparable [6,19].

Beyond modifying payload delivery, nanocarriers can actively interact with payload-induced responses, giving rise to *composite functional fingerprints*. These emergent fingerprints reflect the integration of carrier-driven cellular states with payload-driven perturbations and may amplify, buffer, redirect, or qualitatively transform biological outcomes. Such interactions are frequently non-additive and context-dependent, reflecting system-level responses rather than simple summation of individual components [2,15,24].

Composite fingerprints challenge the assumption that the biological effect of a nanoparticle system can be decomposed into separable carrier and payload contributions. Instead, they suggest that the nanocarrier–payload assembly constitutes a distinct biological entity whose impact emerges from dynamic interactions among material properties, pharmacological activity, and cellular state [2,15,16]. Future efforts may benefit from quantitative modeling approaches capable of describing non-additive interactions between carrier- and payload-driven programs. Conceptual frameworks borrowed from synergy and antagonism modeling could, in principle, be adapted to multidimensional omics signatures, enabling a more formal description of emergent composite fingerprints. This perspective helps explain why closely related nanoparticle formulations delivering the same drug can exhibit divergent efficacy or toxicity profiles across cancer models, and why optimization strategies focused solely on delivery efficiency or drug loading often fail to capture relevant biological complexity [2,15].

Experimentally, distinguishing payload-driven fingerprints from composite fingerprints requires explicit comparison between free payloads, empty nanocarriers, and loaded nanoparticles under aligned conditions. Omics-based approaches are particularly well suited to this task, as they enable pathway-level comparisons across these conditions and reveal non-linear response patterns indicative of emergent behavior [16]. By shifting attention from isolated endpoints to cellular state transitions, functional fingerprinting provides a richer framework for interpreting both therapeutic efficacy and unintended effects.

## 4. Context Dependence and Heterogeneity of Functional Fingerprints

A central challenge in interpreting nanocarrier-, payload-, and composite-driven functional fingerprints is their strong dependence on biological context. Cancer cells occupy diverse functional states shaped by tissue of origin, oncogenic drivers, metabolic configuration, and microenvironmental constraints [25]. Consequently, the same nanotherapeutic system can induce markedly different fingerprints across cancer models, even when physicochemical properties and exposure conditions are nominally matched [4,16].

Context dependence is particularly evident for stress adaptation and metabolic plasticity. Nanocarrier-driven responses that are tolerated in one cellular background may trigger cytotoxic or growth-inhibitory effects in another, while payload-induced perturbations may be buffered or amplified depending on the pre-existing state of proteostasis, redox balance, or nutrient availability [16,26]. In addition, differences in nanoparticle uptake, intracellular trafficking, and effective intracellular dose across cellular states further contribute to divergent functional fingerprints, even under identical external exposure conditions.

Intratumoral heterogeneity further complicates interpretation. Bulk omics measurements necessarily average responses across subpopulations that differ in uptake efficiency, intracellular routing, stress tolerance, and payload sensitivity. As a result, composite fingerprints inferred from bulk data can obscure divergent cellular trajectories, including the coexistence of sensitive, resistant, and adaptive states within the same system [16,27]. These minority populations may ultimately drive treatment failure or delayed toxicity despite favorable average responses.

Microenvironmental factors add an additional layer of complexity. Gradients of oxygen, nutrients, extracellular matrix composition, and interactions with stromal or immune cells influence nanoparticle transport, cellular entry, and functional impact. Nanocarrier–payload systems may therefore elicit distinct fingerprints in hypoxic versus normoxic regions, at invasive fronts versus tumor cores, or in immune-rich versus immune-excluded niches [4,16].

Together, these factors underscore that functional fingerprints are not fixed properties of nanoparticle formulations, but are emergent features shaped by cellular and environmental context.

## 5. Resolving Functional Fingerprints: Higher-Resolution Approaches

The recognition that functional fingerprints are strongly context-dependent and shaped by cellular heterogeneity highlights a fundamental limitation of bulk analyses. While bulk omics approaches have been instrumental in identifying coordinated response programs, they inherently average across diverse cellular states and spatial niches. As a result, bulk analyses obscure the contribution of minority subpopulations and limit the ability to assign functional fingerprints to specific cellular trajectories or microenvironmental conditions [27].

Higher-resolution approaches offer a path to overcome these limitations. Single-cell profiling strategies enable the dissection of heterogeneous responses to nanocarrier–payload systems by resolving cell-to-cell variability in uptake, stress adaptation, and payload sensitivity [28,29]. Rather than producing a single composite fingerprint, these approaches reveal distributions of response states, capturing the coexistence of sensitive, tolerant, and adaptive subpopulations within the same system [30,31]. Such information is critical for understanding why apparently effective nanoparticle formulations may fail to produce durable responses or may select for resistant cellular states [31].

Spatially resolved analyses further refine functional fingerprinting by preserving tissue architecture and contextual cues [32]. Nanotherapeutic systems encounter heterogeneous environments within tumors, including gradients of oxygen, nutrients, extracellular matrix density, and immune cell infiltration. Spatial approaches make it possible to associate functional fingerprints with specific tumor regions or cellular neighborhoods, linking molecular responses to local delivery efficiency, microenvironmental stressors, and cell–cell interactions [4,33,34]. Importantly, spatial functional fingerprints should not be restricted to cells that have directly internalized nanoparticles. Secondary or bystander effects may arise through cytokine secretion, extracellular vesicle release, or metabolic coupling from nanoparticle-positive cells, thereby propagating response programs to neighboring non-uptake cells. Such localized microenvironmental remodeling may contribute substantially to overall tumor-level outcomes and should be considered when interpreting spatial omics data in nanomedicine studies. This spatial dimension is particularly relevant for nanomedicine, where transport barriers and regional accumulation strongly influence therapeutic outcome [4].

Importantly, higher-resolution data should be viewed as a refinement rather than a replacement of bulk functional fingerprints. Bulk profiles provide a useful overview of dominant response programs, while single-cell and spatial approaches deconvolute these programs into their constituent components. Integrating information across resolutions can help distinguish transient or localized perturbations from robust, system-wide state changes and can identify which cellular subsets drive therapeutic benefit or liability [27,31].

From a translational perspective, resolving functional fingerprints at higher resolution enhances both interpretability and predictive potential. By linking nanocarrier, payload, and composite fingerprints to specific cellular states and microenvironmental contexts, these approaches support more rational selection of nanoparticle designs and therapeutic combinations [16,31]. Ultimately, incorporating resolution as a deliberate design parameter will be essential for transforming functional fingerprinting from a descriptive framework into a tool capable of guiding nanomedicine development.

## 6. Future Directions for Functional Fingerprinting

The framework outlined above implies a shift in how nanoparticle studies are designed, interpreted, and compared. If functional fingerprints are to become informative rather than merely descriptive, experimental strategies must explicitly disentangle nanocarrier-, payload-, and composite-driven effects [2,15]. This shift does not require abandoning established nanomedicine paradigms, but rather supplementing them with design principles that prioritize biological resolution and interpretability.

A first principle concerns the analytical separation of components. Studies aimed at characterizing nanoparticle effects should systematically include empty nanocarriers, free payloads, and loaded nanoparticles under aligned exposure conditions. Without this triad, it is difficult to assign observed molecular programs to carrier activity, payload engagement, or emergent interactions [15]. Functional fingerprinting gains its explanatory power precisely from such comparisons, enabling identification of non-additive behaviors that would otherwise remain obscured.

A second principle relates to the dose and exposure metrics. Functional fingerprints should be anchored, where feasible, to biologically meaningful measures such as internalized dose, subcellular localization, or duration of intracellular exposure, rather than to nominal concentrations alone. Because nanocarriers can decouple administered dose from effective cellular perturbation, omics responses should be interpreted in the context of delivery efficiency and intracellular trafficking behavior [35]. Integrating functional fingerprints with quantitative delivery metrics will be essential for improving reproducibility and cross-study comparability.

A third principle concerns resolution and context awareness. As discussed above, bulk fingerprints provide useful summaries of dominant response programs but can mask heterogeneity that is critical for therapeutic outcome. Incorporating higher-resolution analyses at key stages, such as during formulation optimization or failure analysis, can reveal whether favorable average responses arise from uniform effects or from mixtures of opposing cellular states [16,27].

From a broader perspective, functional fingerprinting also calls for a reassessment of how success is defined in nanomedicine. Traditional endpoints such as tumor accumulation, drug loading, or short-term cytotoxicity provide limited insight into long-term biological consequences [36]. In contrast, fingerprints that capture stress adaptation, metabolic reprogramming, or immune modulation may better predict durability of response, resistance emergence, or off-target effects [2,16,20,37].

Looking forward, the maturation of functional fingerprinting will depend on convergence between experimental and computational approaches. As datasets accumulate, comparative analyses and pattern recognition may enable classification of nanocarrier–payload systems based on shared biological response states rather than on material descriptors alone [16]. While fully predictive models remain a longer-term goal, even partial convergence toward biologically grounded classification would represent a significant advance over current empirical approaches.

In this sense, functional fingerprinting should not be viewed as a replacement for existing nanomedicine metrics, but as a complementary layer that connects material design with biological consequences. By embracing the complexity revealed by omics, the field can move toward more rational, context-aware, and ultimately more effective nanotherapeutic strategies. At present, technical complexity, cost, and data integration challenges limit the widespread adoption of these approaches, underscoring the need for strategic rather than exhaustive application.

## 7. Outlook: Toward Predictive and Context-Aware Nanomedicine

The growing application of omics technologies to nanoparticle research has revealed a level of biological complexity that challenges long-standing assumptions in cancer nanomedicine. Rather than behaving as inert carriers of active agents, nanocarrier–payload systems act as integrated biological perturbations whose effects emerge from interactions among material properties, pharmacological activity, and cellular context. Functional fingerprinting provides a conceptual framework to organize this complexity and to translate heterogeneous molecular responses into interpretable biological states. As summarized in Figure 1, integration of multi-layered omics readouts enables functional fingerprints to link nanoparticle design to context-dependent biological outcomes, supporting more predictive and biologically informed nanomedicine strategies.

Looking ahead, the impact of functional fingerprinting will depend on its adoption as an integral component of nanomedicine design rather than as a retrospective analytical tool. As omics datasets expand and experimental strategies become more standardized, it should become increasingly feasible to compare nanotherapeutic systems based on shared response patterns rather than on material descriptors alone. Such biologically grounded classification could help identify design principles that generalize across models and accelerate the prioritization of nanocarriers and payload combinations with favorable therapeutic trajectories.

Ultimately, the value of functional fingerprinting lies in its potential to anticipate biological outcomes. By aligning nanoparticle design with the cellular states they induce, this approach offers a path toward more predictive and context-aware nanomedicine. Integrating physicochemical determinants with multidimensional biological fingerprints may ultimately support more precise matching between nanoparticle formulations and tumor-specific vulnerabilities. Realizing this potential will require continued integration of high-resolution profiling, quantitative delivery metrics, and mechanistic validation, but the framework outlined here provides a foundation for this next phase of development.

## Data Availability

No new data were created or analyzed in this study. Data sharing is not applicable to this article.

## Abbreviations

The following abbreviation is used in this manuscript:

miRNA	microRNA
